# Supplementary material for: Interpretive Qualitative Evaluation Informs Research Participation and Advocacy Training Program for Seniors: A Pilot Study
Source: Healthcare (Basel). 2023 Oct 3;11(19):2679. doi: 10.3390/healthcare11192679 (PMC10572667; doi:10.3390/healthcare11192679)
Supplement: Supplementary file 1 [file healthcare-11-02679-s001.zip › healthcare-2627641-supplementary.pdf]

Supplemental Material File S1: Interview Guide

"Developing a Research Participation Enhancement and Advocacy Training Program for Diverse Seniors" (DREAMS)

Post-DREAMS Focus groups with Scientific Partners

Interviewer: Thank you so much for agreeing to participate in this focus group to assess satisfaction with and success of the DREAMS Project.

1. What were your expectations regarding participation in this project?

   Probes:
   - Probe for whether or not expectations were met. Why or why not.
   - Probe for any unexpected experiences/outcomes and perceptions of whether these experiences/outcomes were positive or negative.

2. What do you think were strengths (or positive aspects) of the program?

   Probes:
   - Probe for perceptions regarding adequacy of education provided.
   - Probe as needed/indicated for any additional perceptions regarding strengths.

3. Did the program have any weaknesses (was there anything bad about the program)? Probe for specifics as needed/indicated.

4. Based on your experiences do you have any recommendations for how we might improve this education program in the future? Probe as needed/indicated.

5. Based on our discussion today, does anyone have any final thoughts? Additional recommendations? Probe as needed/indicated.
